# Histological Effects of PRP and Corticosteroids Following In Situ Repair of Bursal-Sided Partial Rotator Cuff Tears: An Experimental Rat Study

**DOI:** 10.3390/biomedicines14091926

**Published:** 2026-08-27

**Authors:** Ali Ömer Kaya, Yavuz Akalın, Nazan Çevik, Oğuz Çetin, Fatih Türkmensoy, Özgür Avci, Alpaslan Öztürk

**Affiliations:** Department of Orthopedics and Traumatology, Bursa Yüksek İhtisas Health Research Application Hospital, 16310 Yıldırım, Bursa, Türkiye; aliomerkaya321@gmail.com (A.Ö.K.); nazancevik@gmail.com (N.Ç.); droguz91@hotmail.com (O.Ç.); turkmensoyfatih@gmail.com (F.T.); zgravc88@gmail.com (Ö.A.); ozturkalp16@gmail.com (A.Ö.)

**Keywords:** shoulder, rotator cuff, steroid, platelet-rich plasma

## Abstract

**Background/Objectives:** This study aimed to compare the histopathological effects of subacromial corticosteroid (CS) and platelet-rich plasma (PRP) injections following in situ repair of bursal-sided partial supraspinatus tears in a rat model. **Methods:** Twenty-seven Wistar Albino rats were included in the study. Partial bursal-sided supraspinatus tears were surgically created and repaired in situ. Animals were randomly assigned to receive a subacromial injection of 0.6 mL of normal saline (control), methylprednisolone sodium succinate (CS; 4.8 mg/kg, 0.6 mL), or 0.3 mL of activated PRP per rat. After four weeks, the repair sites were harvested for histopathological evaluation of cellular morphology, apoptosis, collagen organization, and vascularity. **Results:** PRP preserved the normal spindle-shaped morphology of tendon cells at the osseotendinous junction, whereas CS induced a rounded cellular morphology (*p* < 0.05). CS significantly increased apoptosis scores (*p* < 0.05), while PRP showed apoptosis scores comparable to those of the control group (*p* > 0.05). Collagen organization was preserved in the PRP group but significantly disrupted following CS administration (*p* < 0.05). Vascularity was higher in the PRP group than in the CS group (*p* < 0.05); however, the vascularity findings should be interpreted cautiously. **Conclusions:** Following in situ repair of bursal-sided partial rotator cuff tears, PRP preserved tendon architecture and was associated with a more favorable histopathological profile than corticosteroids. In contrast, CS adversely affected histopathological characteristics by increasing apoptosis and disrupting cellular morphology and collagen organization. These findings suggest that PRP may represent a biologically favorable adjunct following in situ repair of partial-thickness rotator cuff tears; however, the present histopathological findings do not establish accelerated healing, functional superiority, or clinical benefit, and further clinical studies are needed to determine whether these histopathological differences translate into improved clinical outcomes.

## 1. Introduction

Partial-thickness rotator cuff (RC) tears that do not cause significant functional limitations are generally managed conservatively [1]. Surgical intervention is indicated in patients who fail to respond to nonoperative treatment. Surgical options include debridement, conversion of the partial-thickness tear to a full-thickness tear followed by repair, and transtendinous (in situ) repair [2]. Among these techniques, transtendinous repair has gained increasing attention because it preserves the remaining intact tendon fibers and restores the native tendon footprint without completing the tear. Consequently, this approach is considered biologically advantageous by maintaining residual tendon continuity while promoting tendon-to-bone healing. However, despite these theoretical advantages, several clinical studies have reported a higher incidence of postoperative pain and shoulder stiffness compared with tear completion and repair in selected patients [3,4,5].

Unlike nonoperative tendinopathy or full-thickness rotator cuff repair, the biological environment following in situ repair of a bursal-sided partial-thickness tear is characterized by a unique combination of preserved native tendon fibers, surgically repaired tissue, and an active healing response at the tendon–bone interface. The remaining intact tendon fibers continue to contribute to load transmission while simultaneously undergoing biological remodeling. Consequently, successful healing depends not only on controlling inflammation but also on preserving viable tenocytes, maintaining extracellular matrix integrity, restoring collagen organization, and re-establishing vascular supply. Because biological adjuncts administered during the early healing phase may directly influence these interconnected processes, their effects cannot be assumed to be equivalent to those observed in conservatively treated tendinopathy or after repair of full-thickness rotator cuff tears. Despite these important biological differences, the biological healing process following in situ repair remains insufficiently characterized.

Because excessive inflammation during the early healing phase may impair rehabilitation and functional recovery, several biological adjuncts have been investigated to optimize pain control and tendon healing following rotator cuff repair. Among these, corticosteroids (CS) remain one of the most commonly used agents because of their potent anti-inflammatory effects mediated through inhibition of phospholipase A2 and suppression of multiple inflammatory cytokines [6,7]. However, accumulating experimental and clinical evidence indicates that their effects extend well beyond inflammation control. Corticosteroids have been shown to reduce tenocyte viability, induce apoptosis, inhibit fibroblast proliferation, suppress collagen synthesis, disrupt extracellular matrix remodeling, and ultimately compromise the structural integrity and mechanical properties of the healing tendon [8,9]. Although these effects may provide short-term symptomatic relief, they have also raised concerns regarding delayed tendon healing and inferior tissue quality following rotator cuff repair [10].

In contrast, platelet-rich plasma (PRP) has emerged as a promising biological therapy because it contains a high concentration of autologous growth factors capable of modulating multiple phases of tendon healing [11]. Platelet-derived growth factor (PDGF), transforming growth factor-β (TGF-β), vascular endothelial growth factor (VEGF), insulin-like growth factor-1 (IGF-1), and other bioactive mediators released from activated platelets have been shown to stimulate fibroblast proliferation, enhance angiogenesis, promote collagen synthesis, facilitate extracellular matrix remodeling, and support restoration of normal tendon architecture during healing [12,13]. PRP has also been reported to preserve tenocyte viability, reduce apoptotic activity, and facilitate the formation of more organized collagen fibers, although clinical evidence regarding its efficacy after rotator cuff repair remains inconsistent [14,15].

Although corticosteroids and PRP have been extensively investigated in rotator cuff disease, most previous studies have focused on nonoperative treatment or biological augmentation of full-thickness rotator cuff repair [16]. However, the biological effects of postoperative corticosteroid and PRP administration following transtendinous (in situ) repair of bursal-sided partial-thickness rotator cuff tears remain poorly understood. To our knowledge, no previous experimental study has directly compared the postoperative histopathological effects of these two commonly used biological adjuncts following in situ repair in an experimental model. Therefore, the present study was designed to address this important knowledge gap.

Therefore, the aim of the present study was to compare the postoperative histopathological effects of corticosteroid and PRP administration following in situ repair of experimentally created bursal-sided partial supraspinatus tears in a rat model. Histopathological healing was assessed by evaluating cell morphology, apoptotic cell count, collagen organization, and vascularity. We hypothesized that postoperative corticosteroid administration would adversely affect these histological parameters by promoting apoptotic changes and impairing collagen organization, whereas PRP administration would better preserve tendon architecture, support collagen organization and potentially promote vascularity, resulting in a more favorable histopathological healing response.

## 2. Materials and Methods

This study was conducted with the approval of the Local Ethics Committee for Animal Experiments (Approval Number: 2022–15/04). All surgical procedures were performed by the first author as the primary surgeon, with assistance from the second author. Both authors are certified in laboratory animal research and experienced in experimental animal surgery. Daily care, feeding, and health monitoring of the animals were provided by veterinarians at the research center.

A total of 27 female Wistar albino rats, aged 4 months and weighing 200–250 g, were included in the study. Animals were allocated to three equal-sized experimental groups (*n* = 8 per group) from a homogeneous cohort of female Wistar albino rats of similar age and body weight. No allocation was based on animal characteristics, and all animals were housed under identical environmental conditions throughout the study. Three additional rats were used exclusively as blood donors for PRP preparation.

Wistar albino rats were selected because their shoulder anatomy, tendon healing characteristics, reproducibility in musculoskeletal research, ease of handling, and well-established use in experimental rotator cuff studies make them a suitable model for investigating tendon-to-bone healing [17,18,19]. Their suitability and reproducibility in musculoskeletal experimental research have also been supported by recent studies using Wistar rat disease models [19].

Before commencement of the main experiment, pilot procedures were conducted to standardize the surgical model and familiarize the investigators with the rat shoulder anatomy. The final sample size was determined based on preliminary pilot observations, previously published experimental rotator cuff studies using similar animal models [17,18] and ethical principles governing the use of laboratory animals. In accordance with the Reduction principle of the 3Rs framework, the minimum number of animals considered sufficient to achieve the study objectives was selected while maintaining scientific validity and avoiding unnecessary animal use. Consequently, eight animals were included in each experimental group (24 animals in total).

### 2.1. Surgical Technique

**Figure 1:**
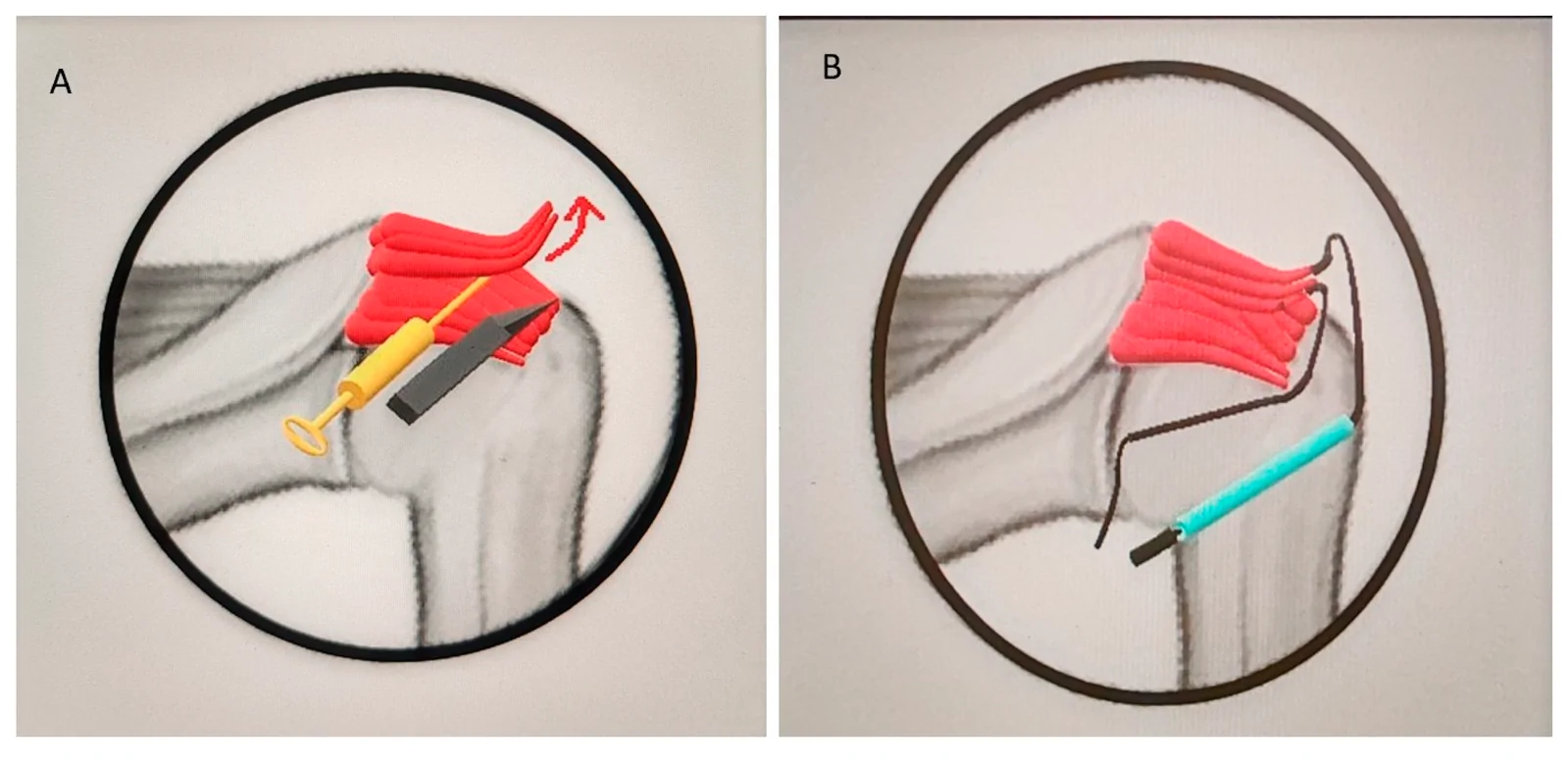
Creation of a partial tear in the supraspinatus osseotendinous junction using a bistoury, as indicated by the arrow pointing towards the bursal region (**A**). Passage of the suture through the tunnel following application of the “Modified Kessler” method to the partial tear and fixation of the rotator cuff to its original insertion site (**B**).

**Figure 2:**
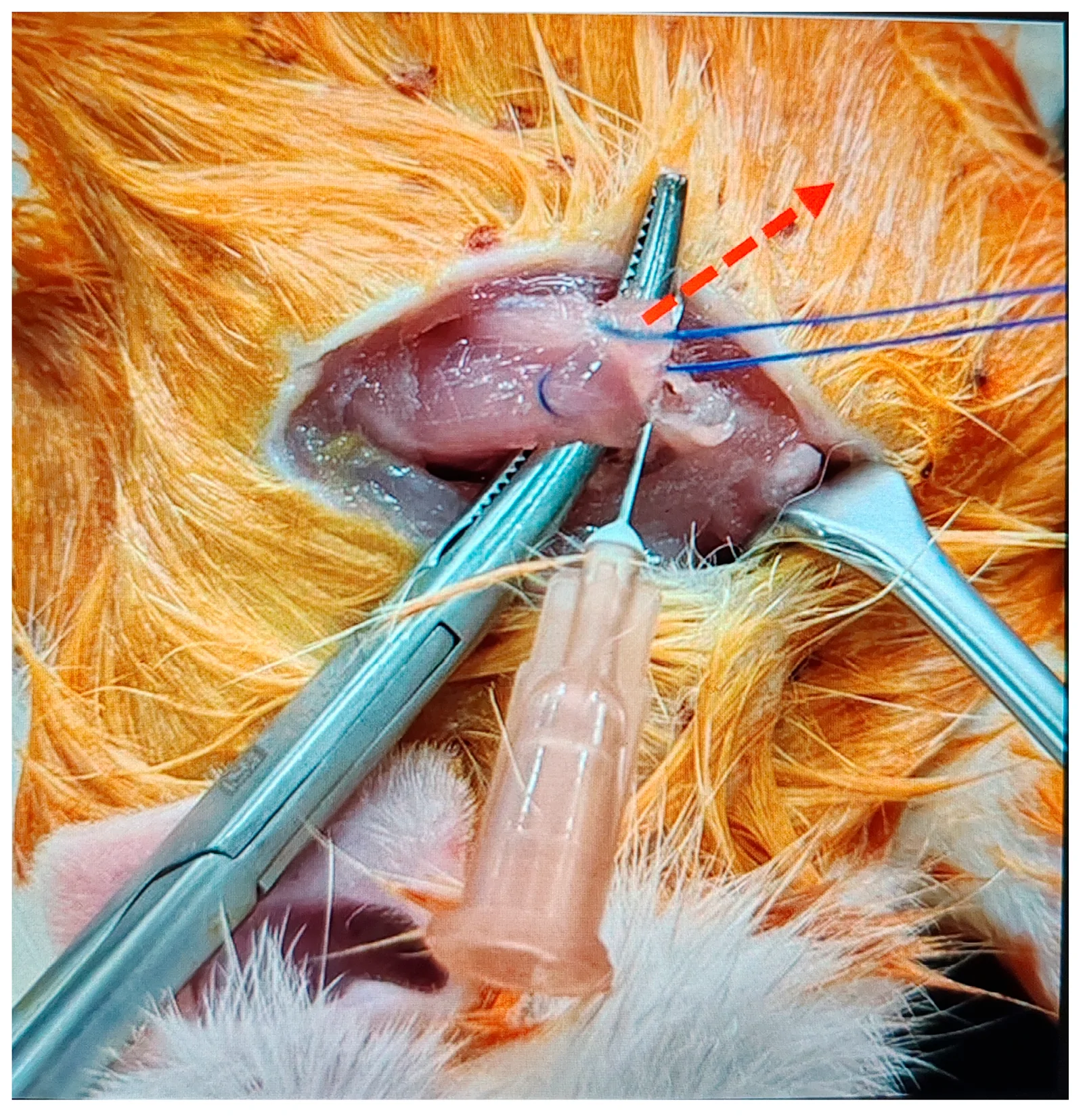
After marking the middle half of the supraspinatus tendon in the sagittal plane with the tip of a dental needle, a bursal-sided partial supraspinatus tear (red dashed arrow) was created by detaching the superior (bursal surface) half from the osseotendinous junction using a scalpel, and it was repaired with a modified Kessler suture technique.

After appropriate anesthesia was administered, a 2 cm incision was made over the acromion. The deltoid was split to expose the rotator cuff. The supraspinatus tendon was exposed, and the midpoint of its insertion was identified using a dental needle as an anatomical reference. A standardized bursal-sided partial-thickness tear was created by sharply detaching the superior half of the supraspinatus tendon from its insertion on the greater tubercle using a scalpel. In all animals, a standardized bursal-sided partial-thickness tear involving the superior 50% of the supraspinatus tendon was created at the midpoint of its insertion using identical anatomical landmarks and surgical instruments to ensure procedural consistency. A 0.5-mm transosseous tunnel was created in the greater tubercle. The detached tendon was repaired using a modified Kessler suture technique with 5-0 Prolene. One limb of the suture was passed through the transosseous tunnel and tied to complete the in situ repair. Following subacromial injection according to group allocation, the deltoid fascia and skin were closed in layers (Figure 1 and Figure 2). Following completion of the repair, a single subacromial injection was administered according to the assigned treatment group before layered closure of the deltoid fascia and skin (Figure 1 and Figure 2). The modified Kessler technique was selected because previous biomechanical studies in rat rotator cuff repair models have demonstrated that it provides a load to failure comparable to the modified Mason-Allen technique while exhibiting lower construct stiffness. Since the primary objective of the present study was to investigate biological tendon healing rather than biomechanical superiority, a repair construct providing adequate initial fixation without unnecessary construct stiffness was considered more appropriate for this experimental model [20].

### 2.2. PRP Preparation

Donor-derived PRP was used to avoid additional blood collection from the experimental animals, thereby preventing blood sampling-related physiological stress and potential hematologic alterations that could influence tendon healing. Furthermore, the use of donor-derived PRP enabled preparation of a standardized PRP product with sufficient volume for all injections. A similar donor-derived PRP protocol has been employed in previous experimental rat rotator cuff studies [21]. Under general anesthesia, whole blood was collected from three donor rats by intracardiac puncture immediately before euthanasia. Approximately 10 ± 1 mL of blood was collected into commercial PRP preparation tubes (T-LAB PRP Tube, 10 mL, 16 × 100 mm; T-LAB, Bursa, Türkiye). Samples were centrifuged at 3200 rpm for 15 min using a Nuve NF 800R centrifuge (Nüve, Ankara, Türkiye). A total of 3 mL of PRP was obtained and activated immediately before injection with 50 μL of 0.55 M calcium chloride (CaCl_2_). Platelet counts were determined by manual counting on Giemsa-stained peripheral blood smears using a light microscope (Olympus BX53, Tokyo, Japan) at ×1000 magnification under immersion oil by two blinded examiners. The baseline whole-blood platelet count of the three donor rats averaged 1.1 × 10^9^ platelets/mL (range, 0.9 × 10^9^–1.25 × 10^9^ platelets/mL). The final PRP platelet concentration was 2.4 × 10^9^ platelets/mL, corresponding to an approximately 2.2-fold platelet enrichment relative to baseline whole blood. Leukocyte concentration was determined using a hemocytometer after dilution with 3% acetic acid and methylene blue and examined under light microscopy (×40 objective), yielding a mean leukocyte count of 30–100 cells/mm^3^, confirming the preparation of leukocyte-poor PRP (P-PRP).

### 2.3. Injections

Immediately after completion of the tendon repair, a single subacromial injection was administered under direct visualization according to group allocation. The subacromial space was identified following deltoid splitting and exposure of the supraspinatus tendon. The control group received 0.6 mL of normal saline, the CS group received methylprednisolone sodium succinate (Prednol-L^®^ (Mustafa Nevzat İlaç Sanayii A.Ş., Istanbul, Türkiye), lyophilized injectable ampoule; 4.8 mg/kg, 0.6 mL), and the PRP group received 0.3 mL of activated PRP per rat. The injection doses and volumes were selected based on previously published experimental rat studies [22,23]. No additional injections were administered during the postoperative period.

Animals were allowed unrestricted cage activity throughout the postoperative period. No animal met the predefined exclusion criteria, including impaired feeding, weight loss, or reduced response to physical stimuli.

The animals were monitored under veterinary supervision for one month after surgery. At the end of this period, all animals were euthanized by cervical dislocation. The humerus, scapula, and rotator cuff tissues were subsequently harvested for histopathological evaluation.

### 2.4. Histopathological Examination

Tissue specimens encompassing the tendon-to-bone repair site were harvested for histopathological evaluation. The specimens were fixed, decalcified in 10% formic acid for 48 h, embedded in paraffin, and sectioned at a thickness of 5 μm. Hematoxylin and eosin (H&E) staining was used to evaluate cellular morphology, apoptotic activity, and vascularity, whereas Masson’s trichrome staining was performed to assess collagen organization.

Histopathological evaluation was performed at the tendon-to-bone repair site. All sections were independently examined by two experienced pathologists who were blinded to group allocation. Histological scoring was performed using a modified Bonar tendon scoring system based on the original method described by Bonar et al. and the modifications proposed by Maffulli et al. [24,25]. Consistent with previous experimental rat rotator cuff studies [26], the modified Bonar scoring system was used to evaluate tendon-to-bone healing. To facilitate histopathological interpretation and provide a more intuitive assessment of tendon healing, the scoring direction was intentionally modified so that higher scores represented more favorable histological findings, whereas lower scores indicated more advanced pathological changes. Four parameters (cellular morphology, apoptosis, collagen organization, and vascularity) were evaluated. Intermediate scores (1.5, 2.5, and 3.5) were permitted when the histological findings were considered to fall between two adjacent grades (Supplementary Table S1). The final score for each specimen was calculated as the mean of the scores assigned independently by the two pathologists and was used for statistical analysis. Interobserver agreement was assessed using linear weighted kappa statistics prior to statistical analysis.

### 2.5. Statistical Analysis

The scores obtained from the histopathological examination were recorded in a data table. Data on cellular morphology, apoptosis, collagen organization, and vascularity were separately analyzed among the control, CS, and PRP groups. Statistical analysis was performed using IBM SPSS Statistics, version 22.0. Overall differences among the three groups were evaluated using the Kruskal–Wallis test. When the Kruskal–Wallis test showed significant differences, pairwise comparisons were conducted using the Mann–Whitney U test. Because tied observations were expected due to the ordinal and discrete nature of the histopathological scoring system, tie-adjusted asymptotic two-sided *p*-values were used for the Mann–Whitney U comparisons. The Kruskal–Wallis test statistics were also adjusted for ties by SPSS. No adjustment for multiple pairwise comparisons was applied; therefore, these post hoc comparisons were considered exploratory and interpreted cautiously. A two-sided *p*-value < 0.05 was considered statistically significant.

Differences between groups in the Mann–Whitney U test were evaluated using the effect size, which was calculated from the standardized Z statistic as r = |Z|/√N, where N represents the total number of observations included in the two groups being compared. Cohen’s criteria were used to interpret the magnitude of the effect size as small (r ≈ 0.10), medium (r ≈ 0.30), and large (r ≥ 0.50). For the Kruskal–Wallis test, effect size was calculated using epsilon squared (ε^2^), according to the formula ε^2^ = (H − k + 1)/(N − k), where H is the Kruskal–Wallis test statistic, k is the number of groups, and N is the total sample size, and its interpretation was based on the following thresholds: values between 0.01 and less than 0.06 indicate a small effect, values between 0.06 and less than 0.14 indicate a medium effect, and values equal to or greater than 0.14 indicate a large effect.

## 3. Results

**Figure 3:**
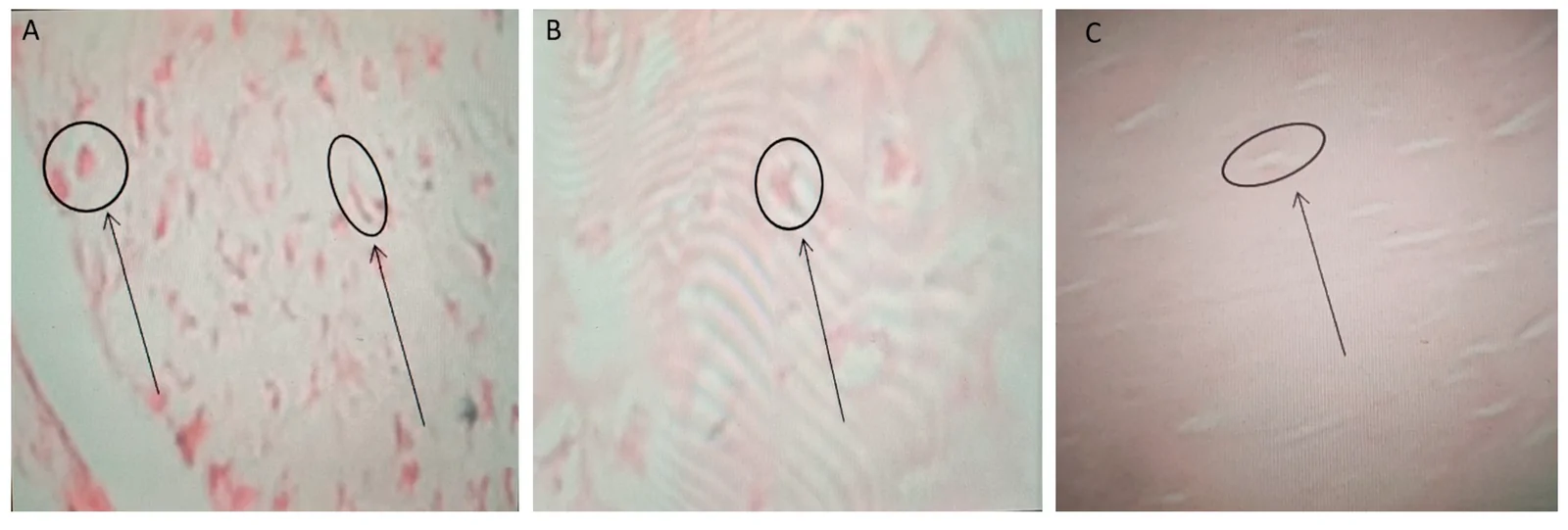
Histopathological examination of cell morphologies (H&E, ×40). (**A**) In the control group, cells exhibiting both spindle-shaped morphology (enclosed in ovals) and a rounded appearance (enclosed in circles) were observed. (**B**) In the CS group, a predominance of rounded cells (enclosed in circles) was observed. (**C**) In the PRP group, a predominance of spindle-shaped cells (enclosed in ovals) was observed. CS: Corticosteroid; PRP: Platelet- rich plasma.

Cellular morphology differed significantly among the three groups (Kruskal–Wallis H = 14.734, *p* = 0.001, ε^2^ = 0.606), indicating a large overall effect. PRP preserved the natural spindle-shaped morphology, whereas CS caused a notable shift toward rounded cell appearance. Pairwise comparisons demonstrated significant differences between the Control and CS groups (*p* = 0.020, r = 0.58), the Control and PRP groups (*p* = 0.020, r = 0.58), and the CS and PRP groups (*p* = 0.001, r = 0.84) (Table 1) (Figure 3).

Apoptosis scores differed significantly among the groups (H = 12.879, *p* = 0.002, ε^2^ = 0.518). The PRP group had the highest scores, corresponding to more favorable histological findings with fewer apoptotic cells, whereas the CS group had the lowest scores. Pairwise analysis demonstrated significant differences between the Control and CS groups (*p* = 0.026, r = 0.56) and between the CS and PRP groups (*p* = 0.001, r = 0.85). The difference between the Control and PRP groups was not statistically significant (*p* = 0.100, r = 0.41) (Table 1).

**Figure 4:**
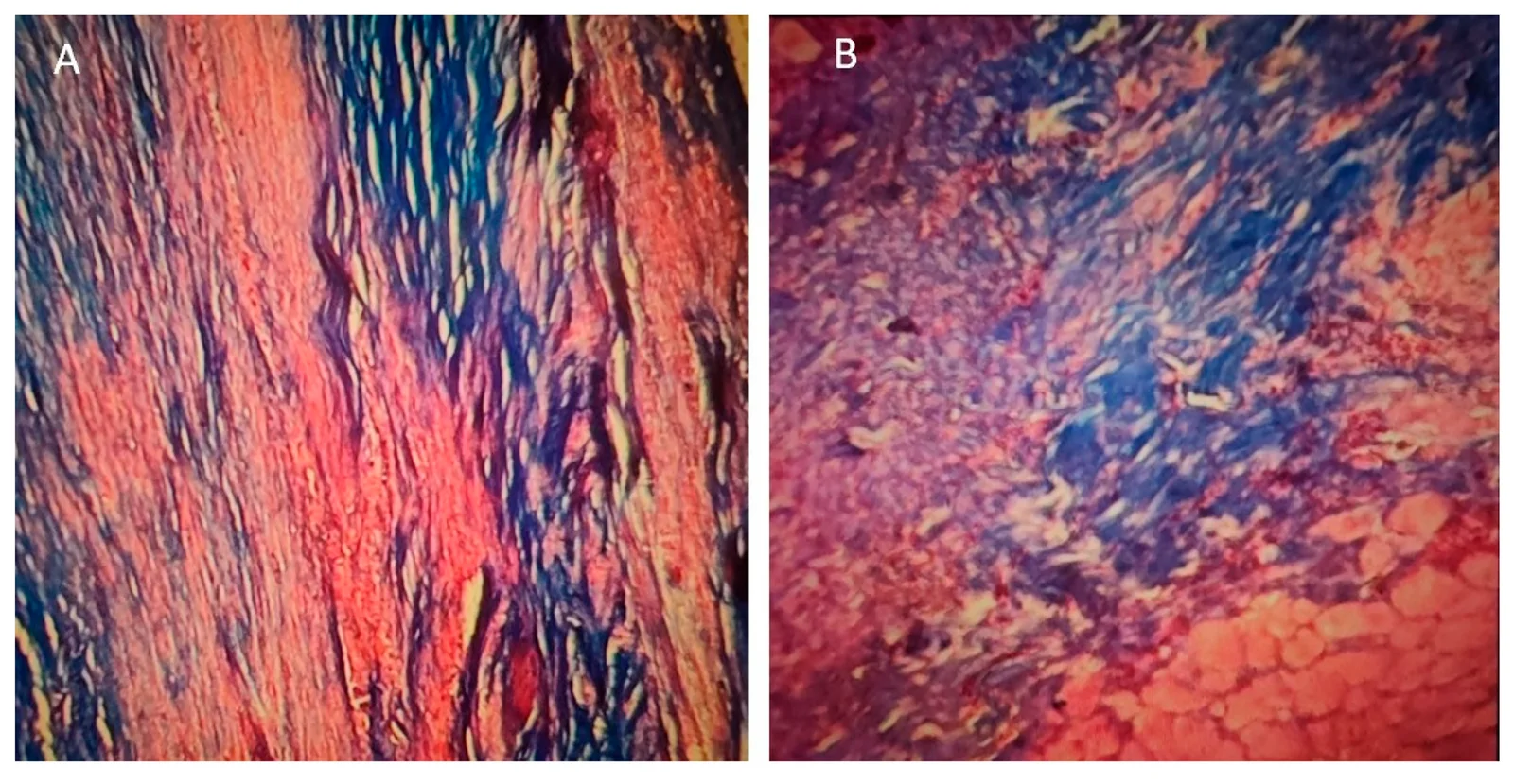
Histopathological examination of collagen arrangement (Masson’s Trichrome staining, ×40). (**A**) Control group; (**B**) CS group. Compared to the regular collagen organization in the control group (**A**), disruption in collagen organization was observed in the CS group (**B**). CS: corticosteroid.

Hematoxylin and Eosin (H&E) staining did not reveal significant differences in collagen alignment among the groups; therefore, further analysis was conducted using Masson’s trichrome staining. Masson’s trichrome scores differed significantly among the groups (H = 8.401, *p* = 0.015, ε^2^ = 0.305), indicating a large overall effect. Pairwise comparisons demonstrated significant differences between the Control and CS groups (*p* = 0.016, r = 0.60) and between the CS and PRP groups (*p* = 0.011, r = 0.64), with lower collagen organization scores in the CS group. No significant difference was observed between the Control and PRP groups (*p* = 0.551, r = 0.15) (Table 1) (Figure 4).

**Figure 5:**
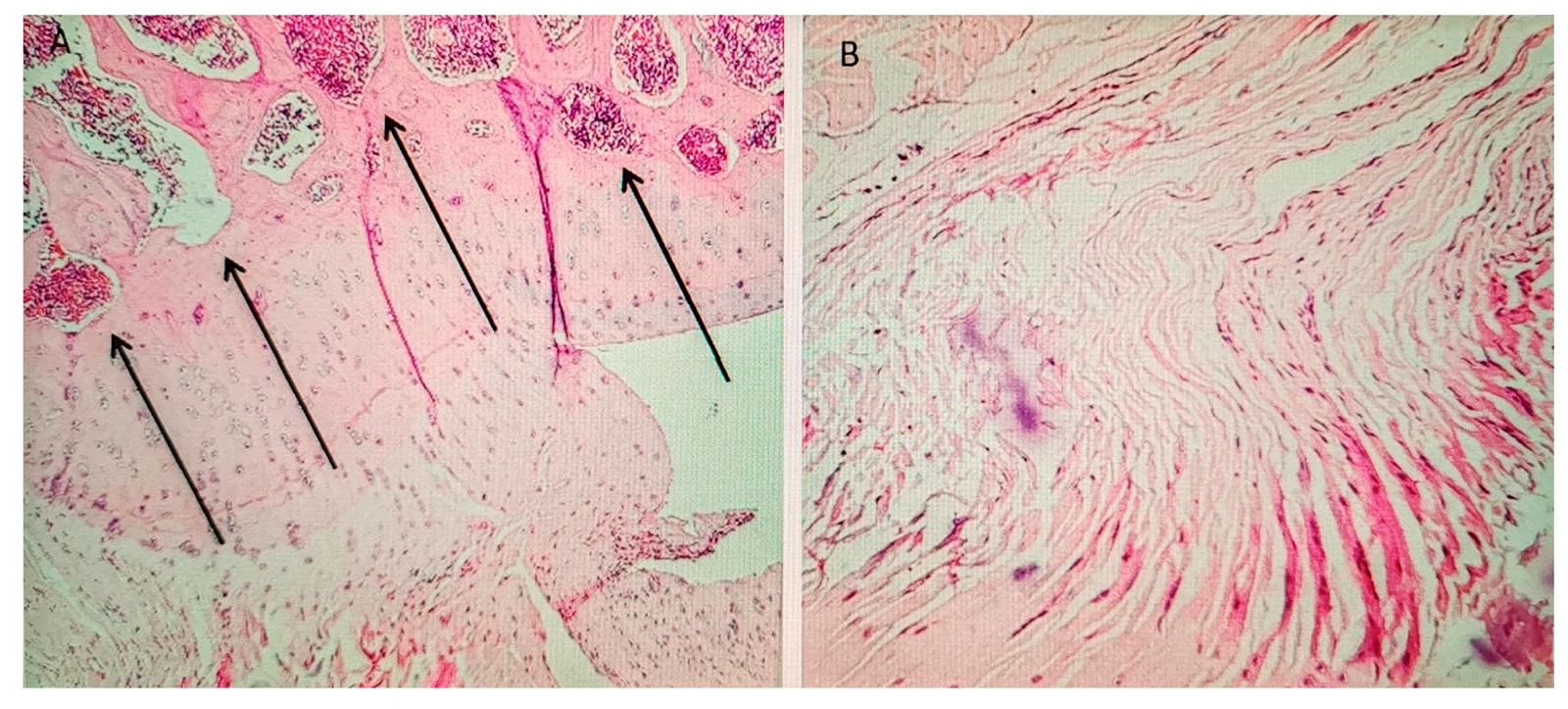
Histopathological examination of vascularization (H&E, ×20). (**A**) An increase in vascularization was observed in the areas marked with an arrow in the PRP group. (**B**) In the CS group, no increase in vascularization was observed. CS: corticosteroid; PRP: platelet-rich plasma.

Vascularity scores at the osseotendinous junction differed significantly among the groups (H = 9.580, *p* = 0.008, ε^2^ = 0.361). The difference between the Control and CS groups was not statistically significant (*p* = 0.183, r = 0.33). The Control–PRP comparison reached nominal statistical significance (*p* = 0.046, r = 0.50); however, given its proximity to the prespecified significance threshold and the absence of adjustment for multiple pairwise comparisons, this finding was interpreted cautiously. The PRP group demonstrated significantly higher vascularity scores than the CS group, with a large effect size (*p* = 0.004, r = 0.72) (Table 1) (Figure 5).

Interobserver agreement between the two blinded pathologists was very good for cellular morphology (linear weighted κ = 0.89), good for apoptosis (κ = 0.61) and collagen organization (κ = 0.67), and moderate for vascularity (κ = 0.46).

## 4. Discussion

In the present study, we evaluated the histopathological effects of postoperative corticosteroid (CS) and platelet-rich plasma (PRP) administration following in situ repair of surgically created bursal-sided partial supraspinatus tears in a rat model. Our findings demonstrated that CS administration was associated with adverse histopathological changes, including altered tenocyte morphology, increased apoptosis, disruption of collagen organization, and reduced vascularity. In contrast, PRP preserved tendon architecture and cellular morphology while promoting a more favorable histopathological healing response. Our findings demonstrated contrasting histopathological responses to CS and PRP administration across cellular morphology, apoptosis, collagen organization, and vascularity. Rather than indicating accelerated healing, these findings reflect differences in the histological characteristics of tendon healing at the evaluated time point.

One of the most important findings of this study was the detrimental effect of CS on tendon cell morphology and viability. Compared with the characteristic spindle-shaped morphology observed in healthy tendon tissue, tenocytes in the CS-treated group exhibited a rounded appearance accompanied by significantly increased apoptosis. Similar findings have been reported in previous experimental studies. Nakamura et al. [27] demonstrated ultrastructural evidence of apoptosis and secondary necrosis in tendon fibroblasts within 24 h after CS administration, whereas Muto et al. [28,29] reported reduced tendon cell viability and increased apoptotic activity following corticosteroid exposure. These morphological and apoptotic changes should not be considered isolated findings, as glucocorticoid-induced cytotoxicity may reduce the viable tenocyte population required for extracellular matrix synthesis and remodeling. Impaired tenocyte viability may consequently compromise matrix turnover and the maintenance of normal tendon architecture during the healing process. Together with our findings, these studies suggest that the adverse effects of corticosteroids on tendon healing may involve direct cytotoxic effects on tendon cells as well as subsequent impairment of extracellular matrix remodeling.

Conversely, PRP preserved the normal spindle-shaped morphology of tendon fibroblasts, while apoptosis scores remained comparable to those of the control group. These findings support the growing body of experimental evidence indicating that PRP may exert cytoprotective effects during tendon healing. Kim et al. [30] demonstrated that PRP suppresses apoptosis by downregulating caspase-3 and HSP-70 expression, thereby facilitating tendon regeneration. Similarly, Takase et al. [31] reported that PRP prevented adipocyte differentiation and preserved normal cellular morphology in a rat rotator cuff model through the action of TGF-β. These effects may be mediated by the coordinated release of platelet-derived growth factors, including platelet-derived growth factor (PDGF), transforming growth factor-β (TGF-β), vascular endothelial growth factor (VEGF), and insulin-like growth factor-1 (IGF-1), which can stimulate tenocyte proliferation, enhance cellular viability, and support tissue repair. However, PRP should not be regarded as a standardized biological product. Its biological activity may vary according to platelet concentration, leukocyte content, activation method, and the timing of administration. Differences in these characteristics may partly account for the heterogeneous effects of PRP reported across experimental and clinical studies. In the present study, leukocyte-poor PRP with a defined platelet enrichment and calcium chloride activation protocol was used, and the findings should therefore be interpreted within the context of this specific PRP preparation.

Masson’s trichrome staining demonstrated poorer collagen organization in the CS group, whereas collagen organization in the PRP group remained comparable to that of the control group. These findings are consistent with previous animal studies reporting collagen fragmentation, matrix disorganization, and tendon degeneration following corticosteroid administration. Akpinar et al. [18] demonstrated fragmentation of collagen fibers after repeated methylprednisolone injections, while Ghellioni et al. [32] and Şahin et al. [33] similarly reported deterioration of collagen architecture following corticosteroid exposure in experimental rotator cuff models. Collagen organization is particularly relevant to tendon repair because the transition from a disorganized provisional matrix toward more regularly aligned collagen fibers represents an important component of extracellular matrix remodeling and tendon maturation. Disruption of tenocyte viability and matrix synthesis by corticosteroids may interfere with this remodeling process and thereby compromise restoration of organized tendon architecture. In contrast, PRP has been shown to improve collagen maturation, promote collagen fiber alignment, and preserve extracellular matrix organization during tendon healing [34]. These biological effects are thought to result from enhanced fibroblast activity and extracellular matrix remodeling, ultimately facilitating tendon maturation. The preservation of collagen architecture observed in the present study is consistent with these proposed biological mechanisms.

Vascularity differed among the groups, with higher scores observed in the PRP group than in the CS group. Platelet α-granules contain numerous angiogenic mediators, including vascular endothelial growth factor (VEGF), platelet-derived growth factor (PDGF), basic fibroblast growth factor (bFGF), and hepatocyte growth factor (HGF), which may stimulate neovascularization and tissue repair [35,36]. The Control–PRP comparison reached only nominal statistical significance and was close to the prespecified significance threshold; moreover, no adjustment for multiple pairwise comparisons was applied. Therefore, the vascularity finding should be regarded as suggestive rather than definitive, and its biological significance remains uncertain. Previous experimental studies have also demonstrated that corticosteroids suppress angiogenesis and cellular migration after rotator cuff repair, whereas PRP may enhance fibrovascular tissue formation during tendon healing [37]. Thus, although the observed vascularity pattern is biologically compatible with the angiogenic properties of PRP, it should not be interpreted as definitive evidence of enhanced angiogenesis or superior tendon healing.

Taken together, the present findings suggest that the contrasting histopathological responses observed after CS and PRP administration may reflect different biological effects on the healing tendon. Corticosteroids may adversely affect healing through reduced tenocyte viability, increased apoptosis, and disruption of extracellular matrix remodeling, whereas PRP may provide a more favorable biological environment through growth-factor-mediated support of cellular viability, collagen organization, and potentially angiogenic activity. Importantly, these findings demonstrate histopathological differences at a single postoperative time point and do not establish accelerated tendon healing. Confirmation of accelerated or functionally superior healing would require serial assessments and complementary functional or biomechanical testing. Accordingly, the present findings support a biological rationale for PRP following in situ repair of partial-thickness rotator cuff tears but should not be interpreted as evidence of functional superiority or clinical benefit in humans [27,31,32,33,37].

This study has several strengths. To our knowledge, it is among the first experimental studies to directly compare the histopathological effects of postoperative corticosteroid and PRP administration following in situ repair of surgically created bursal-sided partial rotator cuff tears, thereby addressing an important gap in the current literature. In addition, a standardized partial-thickness tear model and a reproducible repair technique were used to minimize procedural variability. Finally, multiple histopathological parameters—including cellular morphology, apoptosis, collagen organization, and vascularity—were evaluated using blinded assessment, providing a comprehensive characterization of the biological response to treatment.

Several limitations should also be acknowledged. First, histological evaluation was limited to hematoxylin–eosin and Masson’s trichrome staining. Additional immunohistochemical analyses, including TUNEL staining and extracellular matrix markers, would have provided a more comprehensive assessment of tendon healing. Therefore, the observed histopathological differences cannot establish functional or biomechanical superiority.

Second, the optimal timing for histopathological evaluation in experimental rotator cuff models remains uncertain. Although previous studies have reported different healing patterns at various postoperative time points [38,39,40], the fourth postoperative week was selected based on available experimental evidence. Therefore, evaluation at earlier or later time points may have yielded different findings. Furthermore, evaluation at a single postoperative time point does not establish accelerated healing, which would require serial assessments.

Third, this study investigated surgically created acute partial-thickness rotator cuff tears. Because many clinical rotator cuff tears are chronic and degenerative, caution should be exercised when extrapolating these findings to chronic tendon pathology.

Fourth, only a single postoperative injection of PRP or CS was administered; repeated injections may produce different biological responses and should be investigated in future studies.

Fifth, donor-derived rather than autologous PRP was used, which limits direct clinical translation. Residual red blood cell contamination was not quantified, and manual platelet counting may introduce measurement variability. Furthermore, the findings are specific to the leukocyte-poor PRP preparation used and may not be generalizable to other PRP formulations.

Sixth, the relatively small sample size (n = 8 per group) limits the ability to detect smaller but potentially biologically meaningful differences and increases the risk of type-II error. Therefore, nonsignificant findings should not be interpreted as evidence of equivalence between groups.

Finally, biomechanical and functional assessments were not performed. Therefore, it remains unclear whether the observed histopathological differences are associated with improvements in the mechanical properties or functional performance of the repaired tendon. However, given the very small size of the rat supraspinatus tendon and the limited dimensions of the in situ partial-thickness tear model, the biomechanical strength after repair would likely be influenced predominantly by the suture construct rather than the intrinsic healing characteristics of the tendon. Consequently, biomechanical testing could have yielded artificially similar results among the study groups, potentially masking treatment-related differences and leading to misleading interpretations. Nevertheless, the observed histopathological differences cannot be equated with functional superiority or clinical benefit.

Overall, these limitations indicate that the findings should be interpreted as differential histopathological responses within this experimental model rather than as definitive evidence of accelerated healing, functional superiority, or clinical benefit. Nevertheless, the standardized surgical model and treatment protocol strengthen the internal validity of the present study and provide a reliable experimental platform for evaluating the histopathological effects of biologic and pharmacologic interventions on rotator cuff healing.

## 5. Conclusions

In conclusion, the principal findings of this experimental study can be summarized as follows:

- Postoperative CS administration following in situ repair of bursal-sided partial supraspinatus tears was associated with adverse histopathological changes, including altered cellular morphology, increased apoptosis, and impaired collagen organization.
- PRP preserved cellular morphology and collagen organization, while apoptosis scores remained comparable to those of the control group.
- Vascularity findings were suggestive of a more favorable response with PRP than with CS; however, their biological significance should be interpreted cautiously.
- Overall, these findings support a biological rationale for PRP following in situ repair of partial-thickness rotator cuff tears; however, they do not establish accelerated healing, functional superiority, or clinical benefit. Further translational and clinical studies are warranted.

## Figures and Tables

**Table 1:** Analysis of cell morphology, apoptosis, collagen arrangement and vascularity grades by groups.

Outcome	Control (*n* = 8)		CS (*n* = 8)		PRP (*n* = 8)		Kruskal–Wallis H	*p*	ε^2^	Pairwise Mann–Whitney U Comparisons
	Mean ± SD	Median (IQR)	Mean ± SD	Median (IQR)	Mean ± SD	Median (IQR)				
Cellular morphology	2.656 ± 0.626	2.625 (2.310–3.180)	1.969 ± 0.364	2.000 (1.560–2.250)	3.406 ± 0.442	3.250 (2.620–3.620)	14.734	0.001	0.606	Control vs. CS: *p* = 0.020, r = 0.58 Control vs. PRP: *p* = 0.020, r = 0.58 CS vs. PRP: *p* = 0.001, r = 0.84
Apoptosis	2.875 ± 0.768	2.875 (2.375–3.437)	2.063 ± 0.458	2.125 (1.562–2.437)	3.406 ± 0.326	3.375 (3.060–3.750)	12.879	0.002	0.518	Control vs. CS: *p* = 0.026, r = 0.56 Control vs. PRP: *p* = 0.100, r = 0.41 CS vs. PRP: *p* = 0.001, r = 0.85
Collagen arrangement	2.813 ± 0.704	2.750 (2.500–3.375)	2.313 ± 0.530	2.000 (2.000–2.375)	3.000 ± 0.655	3.000 (2.500–3.500)	8.401	0.015	0.305	Control vs. CS: *p* = 0.016, r = 0.60 Control vs. PRP: *p* = 0.551, r = 0.15 CS vs. PRP: *p* = 0.011, r = 0.64
Vascularity	2.563 ± 0.563	2.500 (2.000–3.000)	2.188 ± 0.458	2.000 (2.000–2.500)	3.188 ± 0.530	3.250 (2.625–3.500)	9.580	0.008	0.361	Control vs. CS: *p* = 0.183, r = 0.33 Control vs. PRP: *p* = 0.046, r = 0.50 CS vs. PRP: *p* = 0.004, r = 0.72

Pairwise *p*-values are tie-adjusted asymptotic two-sided values. Effect size for Mann–Whitney U comparisons: r = |Z|/√N. Kruskal–Wallis effect size: ε^2^ = (H − k + 1)/(N − k). CS: corticosteroid; PRP: platelet-rich plasma; IQR: interquartile range.

## Data Availability

The datasets generated and analyzed during the current study are available from the corresponding author upon reasonable request.

## Supplementary Materials

The following supporting information can be downloaded at: https://www.mdpi.com/article/10.3390/biomedicines14091926/s1, Table S1: Modified Bonar histological scoring system used in the present study.

## Abbreviations

The following abbreviations are used in this manuscript:

RC	Rotator Cuff
CS	Corticosteroid
PRP	Platelet-Rich Plasma
