# Supplementary material for: Histological Effects of PRP and Corticosteroids Following In Situ Repair of Bursal-Sided Partial Rotator Cuff Tears: An Experimental Rat Study
Source: Biomedicines. 2026 Aug 27;14(9):1926. doi: 10.3390/biomedicines14091926 (PMC13604684; doi:10.3390/biomedicines14091926)
Supplement: Supplementary file 1 [file biomedicines-14-01926-s001.zip › biomedicines-4459514-supplementary.pdf]

Supplementary Table S1. Modified Bonar histological scoring system used in the present study

Parameter	Score 1	Score 2	Score 3	Score 4
Cellular morphology	Markedly abnormal morphology with predominantly rounded cells	Moderately abnormal morphology	Mild morphological alterations with predominantly spindle-shaped cells	Normal spindle-shaped tenocyte morphology
Apoptosis	Marked apoptotic activity	Moderate apoptotic activity	Mild apoptotic activity	Minimal or no apoptotic activity
Collagen organization	Severely disorganized collagen bundles	Moderately disorganized collagen bundles	Mild disorganization with predominantly organized collagen bundles	Well-organized, densely packed collagen bundles with normal alignment
Vascularity	Markedly reduced vascularity	Mild vascularity	Moderate vascularity	Normal or increased vascularity

Note: The scoring system was adapted from the modified Bonar tendon scoring system for the purposes of the present study. The scoring direction was intentionally modified so that higher scores represented more favorable histopathological findings, whereas lower scores represented more advanced pathological changes. Intermediate scores (1.5, 2.5, and 3.5) were permitted when the histological findings were considered to fall between two adjacent grades. The final score for each specimen was calculated as the mean of the scores independently assigned by the two blinded pathologists.
